# Expression of NADPH Oxidase (NOX) 5 in Rabbit Corneal Stromal Cells

**DOI:** 10.1371/journal.pone.0034440

**Published:** 2012-04-12

**Authors:** Farhan Rizvi, Tom Heimann, William J. O'Brien

**Affiliations:** 1 Departments of Ophthalmology, Medical College of Wisconsin, Milwaukee, Wisconsin, United States of America; 2 Department of Microbiology/Molecular Genetics, Medical College of Wisconsin, Milwaukee, Wisconsin, United States of America; University of Oklahoma Health Sciences Center, United States of America

## Abstract

**Purpose:**

To determine whether NOX 5 is expressed in rabbit corneal stromal cells (RCSC). NADPH oxidases (NOXes) are enzymes that preferentially use NADPH as a substrate and generate superoxide. Several isoforms of NOXes function as multi-protein complexes while NOX5 and DUOXs do not require the accessory proteins for their activity and possess calcium binding EF hands.

**Methods:**

Human NOX5 primers were used to amplify the rabbit NOX5 by RT-PCR. Amplified product was sequenced to confirm its identity. The protein encoded by the NOX5 was identified by western blot analysis. NOX5 siRNA was used to reduce transcript, protein, and calcium stimulated activity. *In silico* analyses were performed to establish the putative structure, functions, and evolution of rabbit NOX5.

**Results:**

NOX activity was measured in RCSC with NADPH rather than NADH as a substrate. RT-PCR with NOX5 primers amplified 288 bp product using RCSC cDNA, which, when sequenced, confirmed its identity to human NOX5 mRNA. This sequence was used to predict the rabbit (Oryctolagus cuniculus) *NOX5* gene. NOX5 siRNA reduced amounts of NOX5 mRNA in RCSC and reduced ionomycin stimulated superoxide production. A protein of about 65 to 70 kDa encoded by the NOX5 was detected by western blot analysis. *In silico* analysis predicted a putative rabbit NOX5 protein containing 801 amino acids. Motif searches predicted the presence of at least 3 putative EF-hands in N-terminus and a NOX domain in C terminal region.

**Conclusions:**

The data document that the NOX5 gene was expressed in cells of lagomorphs unlike rodents, making the rabbit an interesting model to study NOX5 functions. The activity of the rabbit NOX5 was calcium stimulated, a trait of NOX5 in general. NOX5 may also prove to be a useful genetic marker for studying the taxonomic position of lagomorphs and the Glires classification.

## Introduction

The NADPH oxidase (NOX) family of proteins exists in several isoforms that transport electrons across biological membranes and produce superoxide using NADPH as an electron donor [1], [2]. The NOX family consists of NOX1, NOX2, NOX3, NOX4, and NOX5, DUOX1 and DUOX2 in humans and their homologues or orthologues in mouse, rat, rabbit, fish, drosophila, and *C. elegans* [3], [4]. NOX derived reactive oxygen species (ROS) including superoxide anion (O_2_^.−^) and hydrogen peroxide (H_2_O_2_) have been implicated in several crucial biological functions from host defense to participation in biosynthetic processes in both phagocytic and non-phagocytic cells [2], [3]. In cells such as cardiac myocytes, dermal epithelial cells and kidney epithelial cells, O_2_^.−^ plays roles in apoptosis, senescence, cell proliferation, oxygen sensing, signal transduction, gene regulation and regulation of hormone synthesis [3], [5], [6].

Members belonging to the NOX family contain a ‘NOX domain’ that is comprised of six transmembrane α-helical domains, two hemes, and predicted regions for FAD and NADPH binding [3]. Some of the NOX members are regulated by other proteins that function as subunits such as p22^phox^, p47^phox^, p67^phox^, p40^phox^, and Rac [7], [8]. Various members require different regulatory subunits, for example, NOX1 requires both p22^phox^ and NOXA1 for activation [2], [9]. NOX2 requires p22^phox^, p47^phox^ as an organizer and p67^phox^ as an activator [2], [5], [8]. NOX3 depending on the species may or may not require activator subunits such as p22^phox^, NOXO1 and NOXA1 for its activity [10]. NOX4 is constitutively expressed and requires p22^phox^, however, a study recently reported Poldip2 may function as an activator [11], [12]. NOX4 expression can be regulated by several molecules including cytokines and growth factors such as IGF1 and TGF-β [13], [14], [15]. *NOX5* located on human chromosome 15, does not require regulatory subunits and its activity is regulated by calcium due to presence of N terminal EF-hands containing calcium binding sites [16], [17]. The calcium binding domain undergoes conformational changes in response to Ca^2+^ and is essential for Ca^2+^ to activate NOX5 [18], [19]. NOX5 isoforms or splice variants in humans include NOX5α, β, γ, ∂, and ε or NOX5-S; however, NOX5-S lacks the EF-hand [20], [21]. In humans NOX5 is expressed in tissues such as: spleen, testis, and vascular tissue, cornea, gastrointestinal tract, and various fetal organs [20], [22]. The functional significance of NOX5 is not understood in detail, although it has been implicated in several cancers, wound healing, endothelial cell proliferation and angiogenesis, in platelet-derived growth factor (PDGF)-induced proliferation of vascular smooth muscle cells and in oxidative damage in atherosclerosis [23], [24]. NOX5 is also expressed in human corneal fibroblasts but its function in cornea remains unknown [22]. It is unclear, whether NOX5 is involved in ceramide induced mitochondrial dysfunction and cell death of corneal stromal fibroblasts [25].

**Figure 1:**
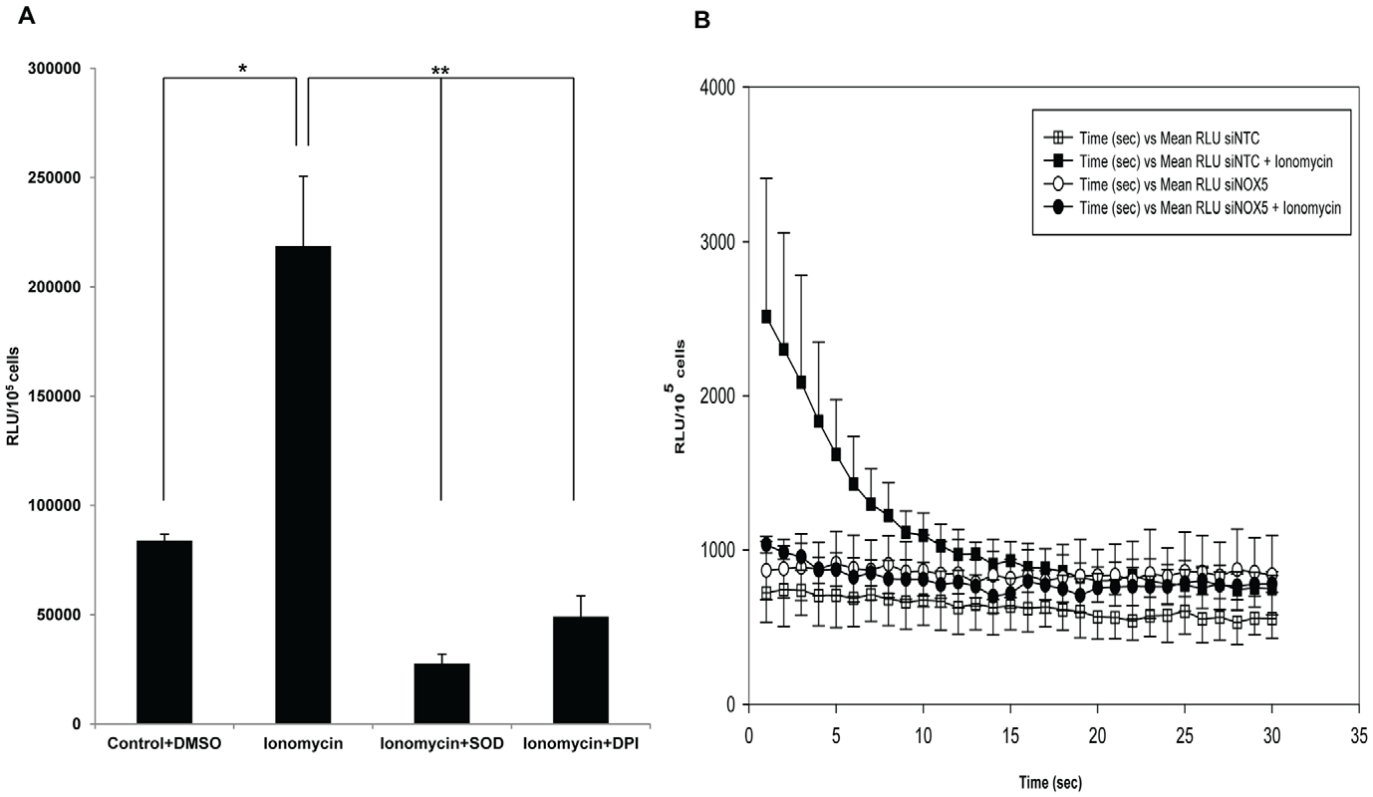
NOX5 regulation and activity in RCSC. (A) Ionomycin stimulated superoxide production (NOX5) was inhibited by SOD and DPI. Harvested RCS cells suspended in Hank's complete with Ca^2+^ were treated with DPI or SOD, as described under Methods. The cells were then stimulated by 100 nM ionomycin or 1 µl DMSO (control). Superoxide production was assayed by chemiluminescence using the Enhanced Diogenes Assay. The area under the curve (AUC) was calculated as a measure of total superoxide production. The values were expressed as RLU/10^5^ cells over the first 30 seconds of the chemiluminescence output. The data represent the mean ± SEM. Asterisks indicate statistical significance: *p<0.05; **p<0.01. (B) RCSC were transfected with either 40 nM non target control siRNA (siNTC) or 40 nM siRNA against NOX5 (siNOX5). After 72 h, the cells were harvested and NOX5 activity was assayed for superoxide production in siNOX5 or siNTC transfectd RCSC with or without ionomycin stimulation. Superoxide production was expressed as RLU/10^5^ cells over the first 30 seconds of the chemiluminescence output. AUC for siNTC with ionomycin (Solid boxes) versus AUC for siNTC without ionomycin (hollow boxes) was statistically significant,*p<0.05.

NOX genes are expressed and proteins producing superoxide have been found in invertebrates and vertebrates [3]. Phylogenetic analyses have predicted the presence of orthologs of NOX5 in both vertebrates and invertebrates such as fish, echinoderms and insects, however; in the order Rodentia, NOX5 was lost during evolution [3]. In the clade Glires, the information that are available regarding NOXes are largely from the studies on rodents; however, reports of the presence of NOX genes and proteins of lagomorphs have been relatively few [26]. Our earlier studies have reported rabbit corneal stromal and epithelial cell cultures express NOX1, NOX4, p22^phox^, p47^phox^, p67^phox^, and p40^phox^ [26]. Other cell types of rabbit origin have also been shown to produce reactive oxygen species (ROS) by various processes reported to be regulated by phorbol 12-myristate 13-acetate (PMA) and Ca^2+^ suggesting the presence of NOX5 [27], [28]. This is the first study to show the presence of NOX5 in lagomorphs. In this study we report a partial mRNA sequence specific to NOX5 as detected in RCSC using human NOX5 primers. *In silico analysis* predicted the putative structure and function of rabbit NOX5, which was also used for studying the evolution of NOX5. The absence of the *NOX5* gene in rodents is one of the major impediments in understanding the physiology of NOX5 in regard to its significance [20], [29]. The presence of the *NOX5* gene in rabbits makes the rabbit a potentially useful model to study *NOX5* gene functions. Its presence in rabbits and its absence in rodents indicates it is a potential genetic marker to study the taxonomic position of Lagomorpha in monophyletic clade Glires [30], [31].

## Materials and Methods

### Cell Cultures

Primary cultures were established from the corneas of New Zealand White rabbits of either sex. The rabbits were obtained from a local vendor and their corneas determined to be free of defects by slit lamp examination. Rabbit corneal stromal cells (RCSC) were isolated as previously described [26], in accordance with the policies stated in the ARVO Statement for the Use of Animals in Ophthalmic and Vision Research and approved by Institutional Animal Care and Use Committee. Briefly, corneal stromal cells were isolated from excised corneas, after removal of the epithelium and endothelium by scraping. The stroma was digested by collagenase. The cells were harvested and grown to confluence at 34°C in DMEM containing 5% FBS, Mito+, and ciprofloxacin. The cells were passaged and were used in the first four passages. In addition to RCSC, human umbilical vein endothelial cells (HUVEC) were obtained from (Clonetics, San Diego, CA; Cat # CC-2517) and initially plated in culture media (Clonetics, Cat# CC-3162). After first passage, the cells were gradually weaned from Clonetics media and grown in M209 (Invitrogen, Cat # 12340), supplemented with 100 U/ml Pen-Strep; 2 mM glutamine, 50 µg/ml EC-mitogen, 100 µg/ml Heparin and 20% FBS. HUVECs were grown at 37 C; 5% CO_2_, and were used in less than 5 passages.

**Figure 2:**
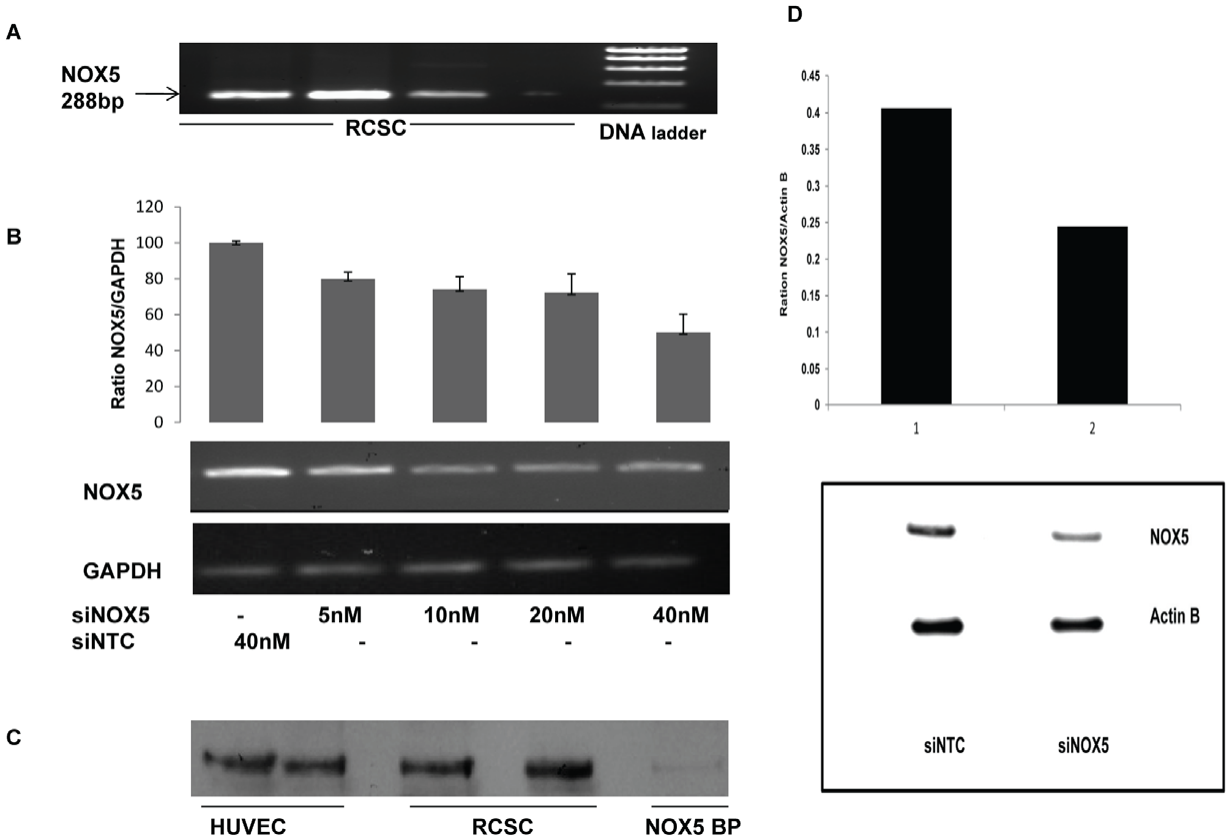
Expression of NOX5 in RCSC determined by RT-PCR and western blot. (A) NOX5 expression detected by RT-PCR using human NOX5-specific PCR primers. The amplified product was confirmed as a NOX5 sequence by nucleotide sequencing (n = 6). (B) Knockdown of the steady state levels of NOX5 mRNA. Values obtained by densitometry were analyzed and ratios were calculated. Amount of knockdown of NOX5 mRNA steady pools are depicted as vertical bars. (1) siNTC (40 nM), (2) siNOX5 (5 nM), (3) siNOX5 (10 nM), (4) siNOX5 (20 nM), (5) siNOX5 (40 nM). (C) Western blot analysis detected by NOX5 specific antibody. RCS cells were grown, harvested, lysed, and fractionated by centrifugation at 29,000× *g*. The pellets were suspended in lysis buffer and subjected to IP for enriching the NOX5 followed by Western blot analysis. Controls included NOX5 encoding HUVEC and anti NOX5 blocking peptide (NOX5 BP). (D) Knockdown of NOX5 determined by Western blotting. RCSC were transfected with 40 nM non target control siRNA (siNTC) or siRNA against NOX5 (siNOX5). After 72 h, the cells were harvested and total protein lysates were used for Western blot analysis by NOX5 specific antibody. β-Actin was used as loading control.

### NOX5 Gene Expression and Sequencing

Reverse Transcriptase Polymerase Chain Reaction (RT-PCR): Total RNA was prepared using Trizol (Invitrogen) followed by DNA digestion with DNase I. cDNA was synthesized using Superscript III (Invitrogen)and used as template for amplification of NOX5. Primers based on the human NOX5 sequence were: forward, *5′- cggtctttcgagtggtttgt-3′* and reverse, *5′- gaagaagacctgcaccttgc-3′*, (NM_024505) from NCBI database. GAPDH primers were used for rabbit GAPDH amplification [32]. The reactions mixtures were prepared using Platinum® Supermix High Fidelity (Invitogen) at 95°C for 5 min followed by 27 cycles (for semi quantitative) or 35 cycles (product amplified for sequencing) of 95°C for 1 min, 56°C for 1 min, and 72°C for 1 min. The 288 bp product was detected on ethidium bromide-stained NuSieve (1.5%) agarose gels. PCR amplified products were prepared for cycle sequencing via gel extraction and purification using a gel extraction kit (Invitrogen). Cycle sequencing of the purified DNA was performed with dye termination sequencing (Prism Big Dye Terminator v3.1 Cycle Sequencing Kit; Applied Biosystems; Foster City, CA). The sequencing reactions were processed on a genetic analyzer (Prism 3100-Avant; Applied Biosystems). The sequence analysis was performed using AB DNA Sequencing Analysis Software v 5.1(Applied Biosystems).

**Figure 3:**
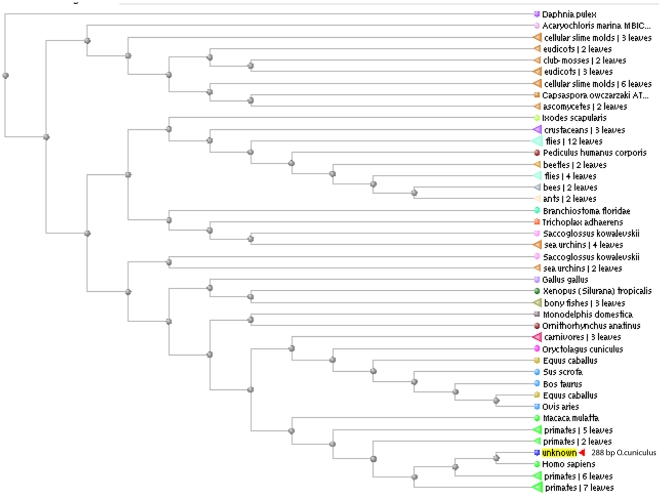
Phylogenetic analysis of NOX5. Phylogenetic analysis of *NOX5* gene based on the schematic phylogeny of organisms created using multiple alignments of the putative amino acid sequence of rabbit NOX5 and deduced amino acid sequences retrieved from the GenBank database. The Tree was reconstructed following NCBI BLAST by the neighbor-joining method. Our rabbit NOX5 sequence (JF723383) is shown as the unknown query.

### NOX5 Immunoprecipitation and Western Blotting

Cell lysates from HUVEC and RCSC were obtained by removing the media and washing the cells with cold PBS followed by treatment with 50 mMTris 0.1 mM EDTA cell lysis buffer (pH = 7.4) containing sodium orthovanadate, PMSF and proteinase inhibitor cocktail (Sigma, St Louis, MO). Cells were lysed by one freeze/thaw cycle and Dounce homogenization followed by sonication (100 Watts for 15 seconds, repeated twice). Cell debris was cleared from the lysates by centrifugation (200× g for 5 minutes at 4°C). The supernatant was collected and further centrifuged at 29,000× g for 30 minutes. The membranes were collected in the form of pellet which was dissolved in 1× RIPA. Protein concentrations were determined using a BCA Protein Assay Reagent Kit (Pierce/Thermo Scientific, Rockford, IL). The proteins from various cell sources were stored at −80°C until their use. Solubilized membrane proteins were enriched by immunoprecipitation (IP) using anti-NOX5 (Cat # sc-34707, Santa Cruz Biotechnology; Santa Cruz, CA)) for overnight treatment followed by adsorption with agarose G beads (20398, Pierce/Thermo, Rockford, IL) for two hours at room temperature. In some studies anti-NOX5 blocking peptide (Cat #sc-34705-P, Santa Cruz Biotechnology) was used as a control. The IP mixtures were centrifuged at 8000× g for 5 minutes and the pellet was washed four times with (1×) washing buffer (Pierce/Thermo, Rockford, IL). The eluted proteins were loaded in NUPAGE 4–12% Bis-Tris Gels (Invitrogen) for western blot analysis to detect NOX5. Proteins were electrophoretically transferred to PVDF filters and were detected using the corresponding horseradish peroxidase-conjugated antibody.

### NOX5 Silencing

Custom made NOX5 siRNA SMARTpool combinations of on target siRNAs were used(Dharmacon, Lafayette, Colorado). The NOX5 siRNA pool consisted of the following siRNA sequences: J-120330-01, NOX5 *CUGCUGAGAAGAAGGGCAA*; J-120330-02, NOX5 *CAAGGUGUUCCAGAAAGU*; J-120330-03, NOX5 *UGGGCAAGAAUGACAUGAA*; J-120330-04 NOX5 *GCUUCCUGGAGCUGCAUAU;* J-010195-06 NOX5 *CUAUAGACCUGGUGACUAC*; J-010195-07 NOX5 *GCGAUUCUUUGCCCUAUUU*; J-010195-08, NOX5 *CCACGUGGCUGGCUCAAGU*; and J-010195-09, NOX5, *CAUCUGCACUGGGCAAGAA*. SiRNA complexes were formed in serum-free medium, using INTERFERIN (PolyPlus, Genesee Scientific; San Diego, CA), according to the manufacturer's instructions. Optimal siRNA/INTERFERIN concentrations were established. Cells grown to about 50% confluence were transfected with 5–40 nM NOX5 siRNA or 40 nM non-target control (siNTC) in DMEM containing 5% FBS without antibiotics for 12 to 16 hr at 34°C. The media was changed and the cells were allowed to grow for additional 72 hours before they were harvested and the RNA isolated and used for RT-PCR.

### Measurement of Superoxide Production in Whole Cells

Low passage RCSC were grown as fibroblasts in DMEM without phenol red containing 5% FBS, Mito+ and ciprofloxacin. Extracellular superoxide generation by whole cells was measured using an enhanced luminol-based chemiluminescence assay reagent, Diogenes (National Diagnostics, Atlanta GA). Harvested cells were collected by centrifugation, washed once in PBS, resuspended in Hanks complete (HBSS with Ca/Mg^2+^), and kept at room temperature until assayed as per the method with slight modifications [1]. In addition cells transfected with 40 nM siNOX5 or 40 nM siNTC were also used to assay ionomycin stimulated superoxide production. For the assay, a 100 µl aliquot of the Diogenes reagent was mixed with a maximum of 0.5×10^5^ cells/100 µl (0.2×10^5^ cells/100 µl for siRNA transfected) in HBSS and incubated at room temperature for 60–90 seconds. Cells were treated with 0.1% DMSO or 100 nM ionomycin. Ionomycin stimulated chemiluminescence (superoxide) was measured every second for up to 5 minutes using a Turner Designs 20/20 luminometer. Area under the curve (AUC) from time first second to 30 seconds (AUC (30s)) was used to determine the superoxide production. Data was analyzed and expressed as RLU (superoxide produced)/10^5^ cells. Statistical analysis was done using the SIGMA Plot 11 (Systat Software Inc, Chicago, IL).

**Figure 4:**
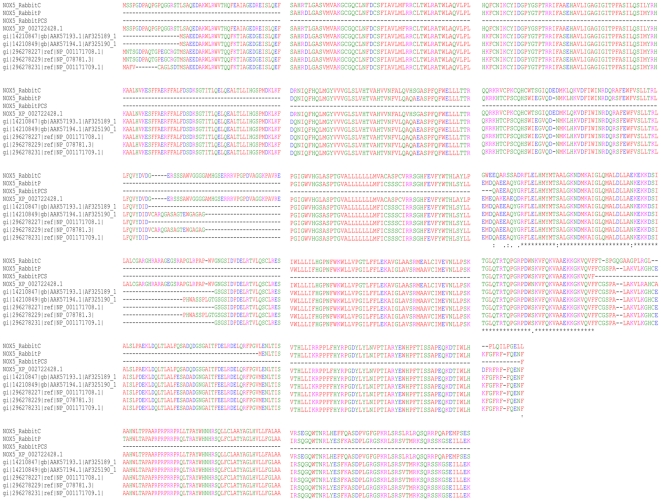
Sequence alignment of rabbit NOX5 with human NOX5 isoforms. Amino acid sequence alignment of rabbit NOX5 (RabbitC (*insilico* cloned), RabbitP (predicted) RabbitPCS (predicted partial) and XP_002722428.1 (predicted NCBI database)) with NOX5 isoforms from human AF325189_1 (isoforms beta), AF325200_1 (isoforms delta), NP_078781.3 (isoforms 1), NP_001171708.1 (isoforms 2), and NP_0011709.1 (isoforms 3) shows rabbit homolog is longer by 18 amino acids.

**Figure 5:**
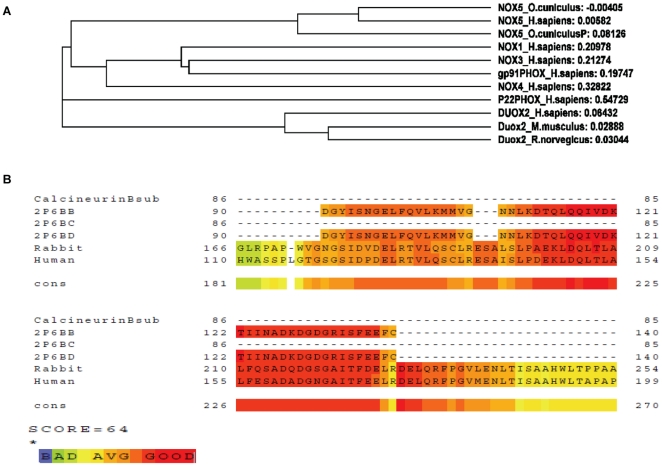
Relationship between rabbit NOX5, Nox/Duox family members, and Calcineurin structure. (5A) Cladogram of sequence relationships among Nox/Duox family members of four species; amino acid alignment of NOX5 from rabbit and human NOX (1–5), human DUOX and Duox from rats and mice was used to construct the cladogram illustrating sequence relationships. Conceptual protein sequences within the regions homologous to putative rabbit NOX5 protein sequence were aligned. Distances show rabbit NOX5 was closer to the human NOX5 than rodent Duox2. (5B) T-coffee analysis of NOX5 amino acid sequence aligning to structure of chain B and D of human Calcineurin ‘B’ subunit structure. 2p6b B confers calcium subunit and may have role in the calmodulin activation of calcineurin.

**Figure 6:**
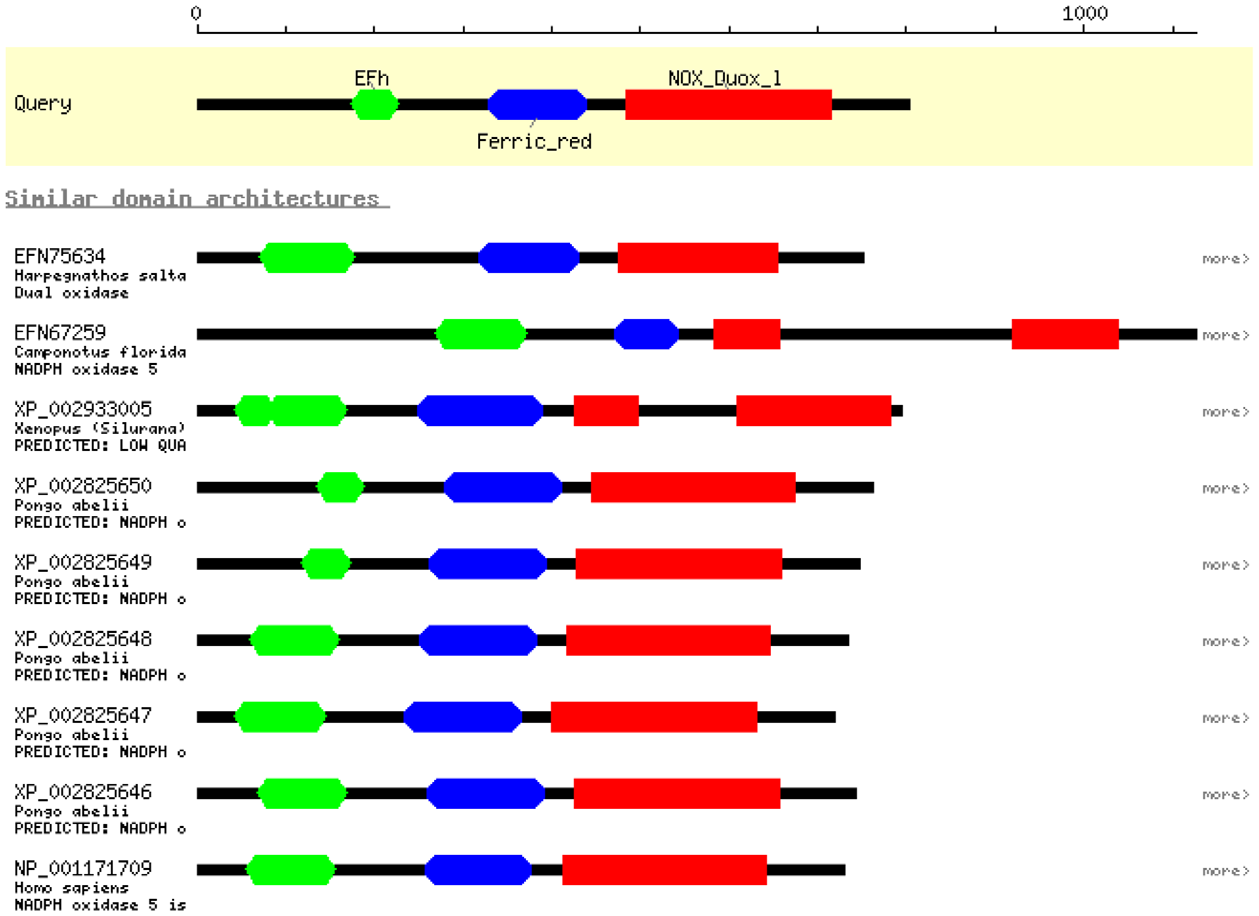
Functional domains and domain architecture based on putative NOX5 sequence. Based on the alignments, the essential functional residues retrieved from the conserved domain architecture retrieval tool (CDART) showed 10 sequences (coelaomata), 88 sequences (Bilateria), 3 sequences (Deuterostomia), 3 sequences (Magnoliphyta), 65 sequences (cellular organisms), and 120 sequences (embryophyta), in addition to several sequences from other organisms predicted to possess domain architectures similar to that of EF hand (cd00051), Ferric reduct superfamily (pfam01794) and NOX_DuoX_1 (cd06186). Figure illustrating few sequences from different species arranged in a bead_on_string_style showing the presence of predicted EF hand (cd00051), Ferric_reduct super family (pfam01794) and NOX_DuoX_1 (cd06186) domains when rabbit NOX5 was used as query sequence for the domain prediction.

### Bioinformatics

The National Center for Biotechnology Information (NCBI) BLAST (http://blast.ncbi.nlm.nih.gov/Blast.cgi) was used to investigate the NOX family of genes. The gene order information was retrieved from Entrez Gene, Ensemble Genome Browser (http://www.ensembl.org/index.html) and Genome Browser (http://genome.ucsc.edu/) from University of California Santa Cruz (UCSC). Partial cDNA sequences were aligned with the predicted genomic NOX5 sequence of rabbit and with human NOX5α in order to obtain the complete sequence. This alignment also permitted us to determine the inferential translational start site of rabbit *NOX5* gene by identifying the open reading frame and Kozak sequence. Sequences of homologues and orthologs of human NOXes and NOX5 isoforms as well as the predicted *O.caniculus* sequence were retrieved from (http://www.ncbi.nlm.nih.gov/gene/) or (http://www.ncbi.nlm.nih.gov/nuccore?term=). The sequence was translated using ExPASy (http://ca.expasy.org/tools/dna.html) and the Cladogram was reconstructed following NCBI BLAST by the neighbor-joining method. Based on their relevance, multiple alignment algorithms programs were used including; multiple sequence alignment and phylogenetic analyses using ClustalW (http://www.ebi.ac.uk/Tools/msa/clustalw2/), COBALT (http://www.ncbi.nlm.nih.gov/tools/cobalt/cobalt.cgi) or T-Coffee (http://www.tcoffee.org/Projects_home_page/t_coffee_home_page.html). CDART (http://www.ncbi.nlm.nih.gov/Structure/lexington/lexington.cgi) and CDD (http://www.ncbi.nlm.nih.gov/Structure/cdd/cdd.shtml) algorithms were used to detect the presence of conserved regions as well as characteristic motifs of the regulatory subunits. The predicted protein structure was generated, compared and assessed using SWISS-MODEL Workspace [33].

## Results

### Superoxide production by RCSC

RCSC were isolated and grown in DMEM supplemented with 5% FBS, Mito +, and ciprofloxacin. Cells were maintained in DMEM without phenol red and other supplements, harvested by trypsinization, treated by soybean trypsin inhibitor and used in assays to detect NADPH dependent, SOD inhibitable O_2_ **^.−^** produced by whole cells. Ionomycin (100 nM) stimulated the O_2_ **^.−^** production by 2.6 fold, which was inhibited by superoxide dismutase (SOD) and diphenyleneiodonium (DPI) (Figure 1A). In siNOX5 transfected cells the production of extracellular superoxide in response to ionomycin was significantly reduced (Figure 1B).

**Figure 7:**
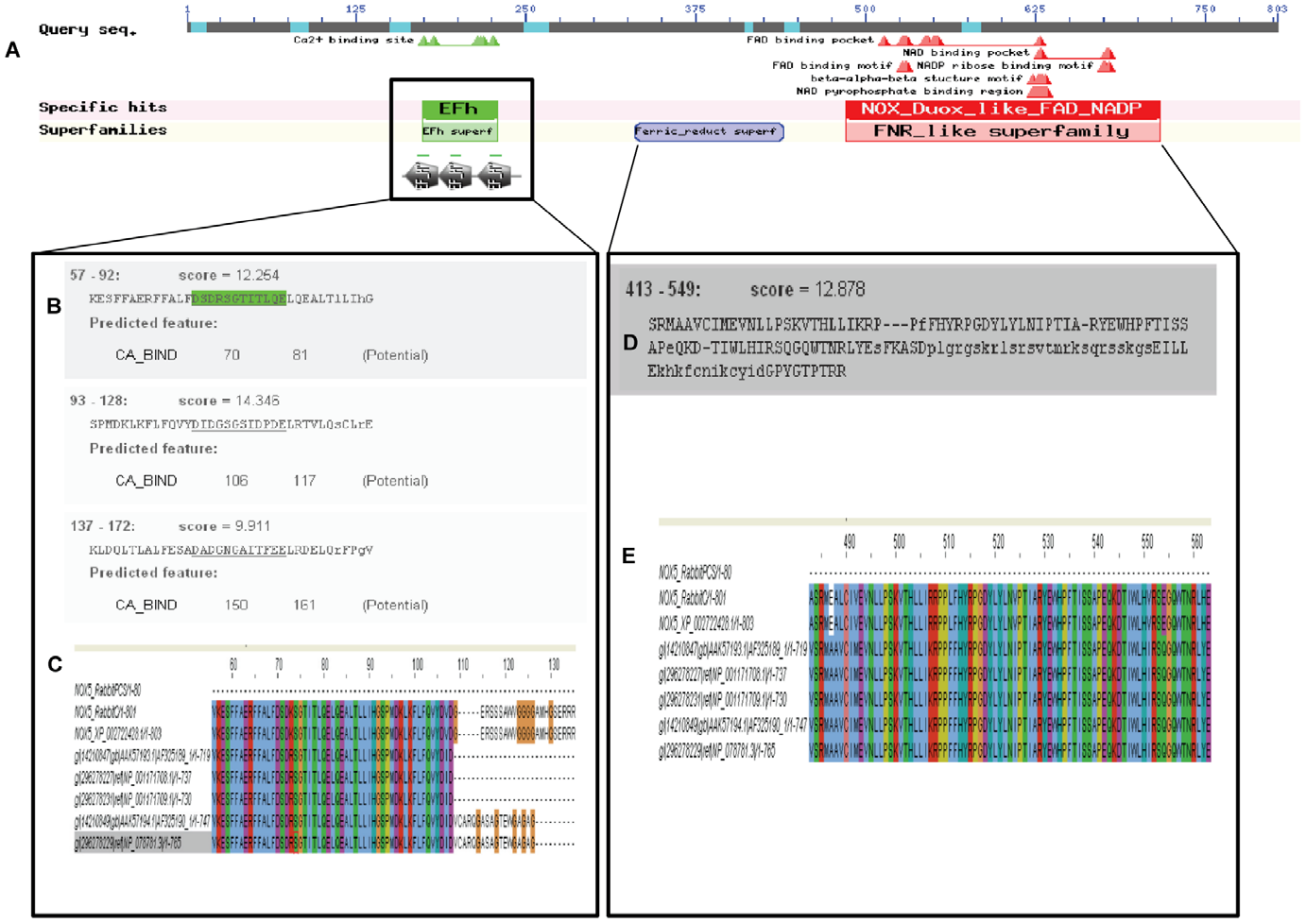
Graphic representations of conserved domains present in the putative rabbit NOX5 sequence. (A) Motif search predicted the presence of at least 3 putative EFh motifs. (B) Predicted EF hands between amino acid 57–92; 93–128 and 137–172 in N-terminal region. (C) Amino acid sequences alignment in the N terminal region showing conserved EFh sequences in rabbit NOX5 and human NOX5 isoforms. (D) NOX/Duox like FAD/NADP binding domain (cd0186) and part of the pfam08030 super family that includes cd06205 and corresponds to Ferrodoxin reductase (FNR), a FAD and NADP binding protein in C terminal region. (E) Conserved sequence of C terminal region highlighting the conserved NOX domain in rabbit and human NOX5 isoforms.

### NOX5 expression in RCSC

RT-PCR using NOX5 primers based on a human sequence (NM_024505) amplified the predicted 288 bp product from rabbit cDNA templates (Figure 2A). The sequence of this product indicated that it was from a partial coding region of a putative rabbit NOX5 mRNA. The BLAST analysis confirmed this product to be a homolog of human *NOX5*. The partial cDNA sequence of rabbit NOX5 was deposited in the GenBank database under the accession number JF723383. We designed siRNAs for NOX5 and transfected RCSC grown in culture to knockdown the NOX5 transcript. The steady state amount of NOX5 mRNA in cells was reduced in cells treated with NOX5 siRNA as determined by RT-PCR (Figure 2B).

A NOX5 antibody (sc-34707) consistently recognized a band of approximately 70 kDa in both NOX5 expressing control cells (HUVEC) and RCSC. A protein enriched by immunoprecipitation with this NOX5 antibody and detected by western blotting appeared to be NOX5 (Figure 2C), since it was blocked by NOX5 blocking peptide (BP) (Figure 2C) and was also showed knockdown by siNOX5 (Figure 2D). Analysis of membrane preparations and cell lysates of RCSC demonstrated a NOX5 antibody reactive protein in the range of 70 to 75 kDa which is in the predicted molecular weight range of NOX5 (65 to 85 kDa).

The 288 bp cDNA sequence on BLASTN showed 98% identity (249/255 bp) to the predicted rabbit NOX5 sequence (XM_002722382.1) and 99% (211/213 bp) to another putative sequence (BR000301.1) of rabbit. In addition it also showed 88% identity (223/252 bp) to human NOX5 (NM_024505.1, NM_024505.3 and gene id 79400) in GenBank. We further used the SPLIGN program, which is usually used for computing cDNA-to-Genomic, or spliced sequence alignments for identifying gene duplication by recognizing introns and splice signals. The partial cDNA sequence (JF723383) using the SPLIGN program was found aligned to human NOX5α in the region of 295755–296925 of NW_001492794.2 (+) and to the predicted rabbit NOX5 sequence XM_002722382.1 in the span of 2077–2331 bp, when it was analyzed against human and predicted rabbit genomic sequences, respectively from the database. We took the help of SPLIGN program to generate a complete predicted sequence of rabbit NOX5 by performing *in silico cloning* where the region of 2077–2331 from predicted sequence of rabbit (XM_002722382.1) was removed and replaced by inserting our RT-PCR generated cDNA sequence (JF723383). Thus, by using ATG of XM_002722382.1 as translation start codon, the full putative translated protein sequence from start to stop codon was obtained. We then used this putative translated sequence obtained as a result of *in silico cloning* for further *in silico* analysis of rabbit *NOX5* gene.

### Evolutionary Importance and Putative Structure of Rabbit NOX5

The hypothetical protein sequence of NOX5 generated as a result of *in silico* cloning was analyzed applying BLAST algorithm. Several hits were identified for the presence of NOX5 across the various phyla, classes, orders and species and few of them are represented as example for the presence of NOX5 in those organisms (Table S1). The phylogenetic tree of NOX5 was created by the neighbor joining method as based on the information retrieved from the GenBank database. Multiple alignments showed that the gene is present in both invertebrates and vertebrates. The gene appeared to be evolved at an early stage of evolution as evident from the presence of sequences similar to NOX5 in members belonging to phyla of echinoderms and arthropods. Among class Mammalia, NOX5 was shown to be present in proximity to genera *Bos*, *Equus*, *Macaca*, *Ovis*, and *Homo*; but, was found absent in genera *Mus sp.* and *Rattus sp.* of rodents (Figure 3).

Comparison between the rabbit and the human NOX5 sequences detected a difference of 18 amino acids in the translation start sites. Nonetheless, most of the regions of NOX5 in human and rabbit showed high level of conservation throughout the coding region (Figure 4). Phylogenetic analysis revealed NOX5 from rabbit and DUOX (1–2) from human, rat and mouse shared homology in their sequences (Figure 5A). In addition to the alignment with NOX5 and other NOXes, we also found that the rabbit sequence showed homology with chain B and D of calcineurin ‘B’ subunit of crystal structure 2p6b (Figure 5B), which is involved in calcium dependent calmodulin activation of calcineurin. The conserved domain architectural retrieval tool (CDART) revealed the presence of an EF-hand (EFh) domain and NOX domain (Figures 6 and 7A), which showed identity across the species (Figure 6). Furthermore, the domain search confirmed the presence of EFh domain (cd00051), a member of EFh calcium binding motif; belonging to the super family cl09501 known to be involved in calcium signaling. The motif search predicted the presence of at least 3 putative EFh motifs in the region between amino acid 57–92; 93–128 and 137–172 in N-terminal region (Figure 7A, 7B) and a NOX Duox like FAD/NADP binding domain (cd0186) which is part of the pfam08030 superfamily that includes cd06205 and corresponds to Ferrodoxin reductase (FNR), a FAD and NADP binding protein in C terminal region (Figure 7D). The domains remained highly conserved in both human and rabbit NOX5 (Figure 4 and 7C, 7E).

## Discussion

The data presented here has identified a rabbit homologue of *NOX5*. We have amplified the *NOX5* gene product by RT- PCR, detected a NOX5 encoded protein by western blotting, measured superoxide production, silenced NOX5 using siRNA designed based on the sequence identified, predicted a rabbit NOX5 sequence, and compared rabbit and human NOX5 mRNA sequences. Our principal findings are that rabbit *NOX5* is a close homologue of the human *NOX5* gene. We determined that ionomycin stimulated O_2_ **^.−^** production by 2.6 fold and was DPI and SOD inhibitable. In the cells transfected with siNOX5, ionomycin failed to stimulate O_2_ **^.−^** production; thus, indicating that the extracellular superoxide was produced by NOX5 in RCSC. The fact that we also observed O_2_ **^.−^** production by non-permeabilized whole cells (Data not shown) and we detect NOX5 protein in membrane preparations further suggested that NOX5 may reside in the cytoplasmic membrane. It was likely responsible for the extracellular O_2_^.−^ [1]. Our previous studies have documented the presence of NOX1 and 4, which would be expected to produce intracellular O_2_ **^.−^** but not extracellular O_2_ **^.−^** [26]. Our analyses suggest that the primary structure of the EF hands and the NOX/DUOX like FAD/NADP binding region as predicted by CDART analysis remain largely conserved in the putative structure of rabbit NOX5 and human NOX5. Members of NOX family are proteins that have preserved the function of transporting electrons across biological membranes to reduce oxygen to superoxide [2]. The conserved structural sequences as observed in the putative rabbit NOX5 are also present in the NOX/DUOX family of human proteins. These structural features determine the enzymatic properties specific to NOX family members (Figure 5B).

Synteny and phylogenetic analyses further suggested that the *NOX5* originated from the common eukaryotic ancestor in the Cambrian period of Paleozoic era as evident from the sequences similar to NOX5 found in the close vertebrate ancestors, the echinoderms [34]. However, this gene was lost in rodents during the course of evolution, perhaps by deletion or mutation that resulted in the degradation of its product beyond recognition. This phenomenon is not unique for NOX5, for example: certain pseudo genes, members of ATP binding cassette (ABC) transporter gene superfamily, CYP1D, and many more genes have been reported to be present in one but lost in other species [35], [36]. Motif searches predicted the presence of three EF hands in the N terminus and a NOX domain in C terminal region in the putative translated sequence of rabbit (Figures 6 and 7A). During the course of evolution, NOX5 acquired EF-hands that made its activity dependent on calcium in contrast to other family members that required regulatory subunits for their activation [3], [20]. Evidence pointed to the presence of single EF hand motif in the members of kingdom *Protista* as the possible precursor of paired EF hands observed in early eukaryotes such as fungi [37], [38]. What prompted the association of Efh domain to NOX5 is not clear. This domain may have been developed as an oxygen sensor to serve as a coupling device to counteract the oxygen toxicity during the Cambrian period. Bivalent calcium may act as messenger binding to the protein causing oxygen to be release in the reduced form [37]. During the early stages of evolution, the oceanic H_2_S/Fe^2+^ buffer system was exhausted due to the rise in the atmospheric oxygen caused by the photosynthesis resulting in a change in the marine redox state. Thus a need existed for the development of well organized and differentiated and compartmentalized cells in eukaryotes [37], [39]. In addition, this rise in oxygen level modified proteins that favored calcium binding thus acting as the sensors of calcium messages [37]. In this way the association of EF hand domains with NOX5 made its activity dependent on calcium levels. It appears that the Ca^2+^- binding domain of NOX5 as an independent folding unit, undergoes conformational changes in response to increase in Ca^2+^ levels and perhaps activates the enzyme through an intramolecular protein-protein interaction between the Ca^2+^-binding region and the enzymatic C terminus facilitating the release of reduced oxygen [18], [19]. Based on these observations it may be presumed that *NOX5* was the first gene to evolve among the Nox/Duox family due to its primitive association and dependence on EF hands. As the dynamic biological system was further differentiated the co-evolution of more regulatory and accessory proteins involved in Nox activity may have interacted later [4]. Hypothetically, as evolution preceded the dependence on EF hands to regulate redox potential was minimized thus resulting in degradation of Efh domains from the NOX/Duox protein family. Notably, one isoform of human NOX5-S lacks EF hands was regulated by Rac1 instead of Ca^2+^ may be the precursor of the later evolved NOXes [40]. It has been proposed that Nox1, Nox2, Nox4 in higher echinoderms, urochordates and teleosts evolved during this later time period [3].

Calcium is essential for NOX5 activity, but in cells of human origin, phosphorylation of NOX5 at T494 and S498, can modify the calcium sensitivity [27]. In this regard, we found NetPhos2.0 (http://www.cbs.dtu.dk/cgi-bin/nph-webface?jobid=netphos,4D938E800409D10E&opt=none) predicted the presence of 18 phosphorylation sites at serine residues, 4 each at threonine, and tyrosine residues in the putative rabbit NOX5 protein (data not shown). In humans, NOX5 has been reported to exist in any of five isoforms (α, β, γ, δ, and ε) [21], which encode proteins of molecular weights from 65 to 85 kDa that vary in the extent of the glycosylation [41]. We are not aware of the presence of multiple NOX5 isoforms in rabbits. Our primers would be expected to amplify all known isoforms. We have identified a NOX5 encoded protein that has a molecular weight between 65 to 85 kDa [41]. The fact that NOX5 siRNA reduced the steady state NOX5 mRNA pools, NOX5 protein and knocks down the ionomycin stimulated O_2_^−^ production in RCSC indicated the activity was due to NOX5. Previously, we showed the constitutive expression of the NOX1 and NOX4 genes as well as production of five accessory proteins necessary to form active NADPH oxidase complexes in cultured rabbit corneal epithelial and stromal cells [26]. Now we have added NOX5 to the repertoire of the NOX family of genes to consider as source of O_2_^−^ in rabbit corneal stromal cells. The functions of the O_2_^−^ produced by each of the NOX oxidases remain to be discovered. Due to the structural similarity to human NOX5, it is assumed that the accessory proteins are not required for the activity of rabbit NOX5. Studies are underway to determine whether any of the NOXes function in the signaling processes of cell death and differentiation of corneal stromal cells. The absence of NOX5 in rodents is an obstacle to studies of the functional significance of NOX5 [20], [29]. This study showing the presence of a *NOX5* gene in rabbits may assist in determining the physiological role of NOX5 and in further understanding the functional relevance of NOX5 in general. In addition, presence of NOX5 in rabbit may be useful genetic marker for positioning Lagomorpha and Rodentia in the clade Glires.

## Supporting Information

Table S1 BLAST analysis revealed the widespread presence of NOX5 across the various phyla, classes, orders, and species.
(PDF)

## References

[1] Kawahara T, Lambeth JD (2008). Phosphatidylinositol (4, 5)-bisphosphate modulates Nox5 localization via an N-terminal polybasic region.. Mol Biol Cell.

[2] Bedard K, Krause KH (2007). The NOX family of ROS-generating NADPH oxidases: physiology and pathophysiology.. Physiol Rev.

[3] Kawahara T, Quinn MT, Lambeth JD (2007). Molecular evolution of the reactive oxygen-generating NADPH oxidase (Nox/Duox) family of enzymes.. BMC Evol Biol.

[4] Kawahara T, Lambeth JD (2007). Molecular evolution of Phox-related regulatory subunits for NADPH oxidase enzymes.. BMC Evol Biol.

[5] Ushio-Fukai M (2006). Localizing NADPH oxidase-derived ROS.. Sci STKE.

[6] Lyle AN, Griendling KK (2006). Modulation of vascular smooth muscle signaling by reactive oxygen species.. Physiology (Bethesda).

[7] Hordijk PL (2006). Regulation of NADPH oxidases: the role of Rac proteins.. Circ Res.

[8] Nauseef WM (2004). Assembly of the phagocyte NADPH oxidase.. Histochem Cell Biol.

[9] Ambasta RK, Schreiber JG, Janiszewski M, Busse R, Brandes RP (2006). Noxa1 is a central component of the smooth muscle NADPH oxidase in mice.. Free Radic Biol Med.

[10] Lambeth JD, Kawahara T, Diebold B (2007). Regulation of Nox and Duox enzymatic activity and expression.. Free Radic Biol Med.

[11] Zhang L, Nguyen MV, Lardy B, Jesaitis AJ, Grichine A (2011). New insight into the Nox4 subcellular localization in HEK293 cells: first monoclonal antibodies against Nox4.. Biochimie.

[12] Lyle AN, Deshpande NN, Taniyama Y, Seidel-Rogol B, Pounkova L (2009). Poldip2, a novel regulator of Nox4 and cytoskeletal integrity in vascular smooth muscle cells.. Circ Res.

[13] Basuroy S, Tcheranova D, Bhattacharya S, Leffler CW, Parfenova H (2011). Nox4 NADPH oxidase-derived reactive oxygen species, via endogenous carbon monoxide, promote survival of brain endothelial cells during TNF-α-induced apoptosis.. Am J Physiol Cell Physiol.

[14] Martin-Garrido A, Brown DI, Lyle AN, Dikalova A, Seidel-Rogol B (2011). NADPH oxidase 4 mediates TGF-β-induced smooth muscle α-actin via p38MAPK and serum response factor.. Free Radic Biol Med.

[15] Meng D, Lv DD, Fang J (2008). Insulin-like growth factor-I induces reactive oxygen species production and cell migration through Nox4 and Rac1 in vascular smooth muscle cells.. Cardiovasc Res.

[16] Bánfi B, Molnár G, Maturana A, Steger K, Hegedûs B (2001). A Ca(2+)-activated NADPH oxidase in testis, spleen, and lymph nodes.. J Biol Chem.

[17] Cheng G, Cao Z, Xu X, van Meir EG, Lambeth JD (2001). Homologs of gp91phox: cloning and tissue expression of Nox3, Nox4, and Nox5.. Gene.

[18] Banfi B, Tirone F, Durussel I, Knisz J, Moskwa P (2004). Mechanism of Ca^2+^ activation of the NADPH oxidase 5 (NOX5) J Biol Chem.

[19] Tirone F, Radu L, Craescu CT, Cox JA (2010). Identification of the binding site for the regulatory calcium-binding domain in the catalytic domain of NOX5.. Biochemistry.

[20] Fulton DJ (2009). Nox5 and the regulation of cellular function.. Antioxid Redox Signal.

[21] BelAiba RS, Djordjevic T, Petry A, Diemer K, Bonello S (2007). NOX5 variants are functionally active in endothelial cells.. Free Radic Biol.

[22] O'Brien WJ, Heimann T, Rizvi F (2009). NADPH oxidase expression and production of superoxide by human corneal stromal cells.. Mol Vis.

[23] Sedeek M, Hébert RL, Kennedy CR, Burns KD, Touyz RM (2009). Molecular mechanisms of hypertension: role of Nox family NADPH oxidases.. Curr Opin Nephrol Hypertens.

[24] Ushio-Fukai M, Nakamura Y (2008). Reactive oxygen species and angiogenesis: NADPH oxidase as target for cancer therapy.. Cancer Lett.

[25] Rizvi F, Heimann T, Herrnreiter A, O'Brien WJ (2011). Mitochondrial dysfunction links ceramide activated HRK expression and cell death.. PLoS One.

[26] O'Brien WJ, Krema C, Heimann T, Zhao H (2006). Expression of NADPH oxidase in rabbit corneal epithelial and stromal cells in culture.. Invest Ophthalmol Vis Sci.

[27] Jagnandan D, Church JE, Banfi B, Stuehr DJ, Marrero MB (2007). Novel mechanism of activation of NADPH oxidase 5 calcium sensitization via phosphorylation.. J Biol Chem.

[28] Weissmann N, Kuzkaya N, Fuchs B, Tiyerili V, Schäfer RU (2005). Detection of reactive oxygen species in isolated, perfused lungs by electron spin resonance spectroscopy.. Respir Res.

[29] Bedard K, Jacquet V, Krause KH (2012). NOX5: from basic biology to signaling in disease. Free Radic. Biol. Med. Free Radic Biol Med..

[30] Ye J, Nie W, Wang J, Su W, Jing M (2011). Genome-wide comparative chromosome map between human and the Forrest's pika (Ochotona forresti) established by cross-species chromosome painting: further support for the Glires hypothesis.. Cytogenet Genome Res.

[31] Asher RJ, Meng J, Wible JR, McKenna MC, Rougier GW (2005). Stem Lagomorpha and the antiquity of Glires.. Science.

[32] Kullberg M, Nilsson MA, Arnason U, Harley EH, Janke A (2006). Housekeeping genes for phylogenetic analysis of eutherian relationships.. Mol Biol Evol.

[33] Arnold K, Bordoli L, Kopp J, Schwede T (2006). The SWISS-MODEL Workspace: A web-based environment for protein structure homology modelling.. Bioinformatics.

[34] He J, Deem MW (2010). Hierarchical evolution of animal body plans.. Dev Biol.

[35] Dean M, Annilo T (2005). Evolution of the ATP-binding cassette (ABC) transporter superfamily in vertebrates.. Annu Rev Genomics Hum Genet.

[36] Kawai YK, Ikenaka Y, Fujita S, Ishizuka M (2010). The CYP1D subfamily of genes in mammals and other vertebrates.. Mamm Genome.

[37] Williams RJP (2006). The evolution of calcium biochemistry.. Biochem Biophys Acta.

[38] Kawasaki H, Nakayama S, Kretsinger RH (1998). Classification and evolution of EF-hand proteins.. Biometals.

[39] Saltzman MR, Young SA, Kump LR, Gill BC, Lyons TW (2011). Pulse of atmospheric oxygen during the late Cambrian.. Proc Natl Acad Sci U S A.

[40] Hong J, Resnick M, Behar J, Wands J, Delellis RA (2011). Role of RAC1 in Regulation of NOX5-S Function In Barrett's Esophageal Adenocarcinoma Cells. Am J Physiol Cell Physiol..

[41] Serrander L, Jaquet V, Bedard K, Plastre O, Hartley O (2007). NOX5 is expressed at the plasma membrane and generates superoxide in response to protein kinase C activation.. Biochimie.

