# Supplementary material for: Expression of NADPH Oxidase (NOX) 5 in Rabbit Corneal Stromal Cells
Source: PLoS One. 2012 Apr 12;7(4):e34440. doi: 10.1371/journal.pone.0034440 (PMC3325249; doi:10.1371/journal.pone.0034440)
Supplement: Table S1 — BLAST analysis revealed the widespread presence of NOX5 across the various phyla, classes, orders, and species. (PDF) [file pone.0034440.s001.pdf]

Accession ID	Gene [Species]
AAK57193.1	NADPH oxidase 5 beta [Homo sapiens]
NP_078781.3	NADPH oxidase 5 isoform 1 [Homo sapiens]
NP_001171709.1	NADPH oxidase 5 isoform 3 [Homo sapiens]
NP_001171708.1	NADPH oxidase 5 isoform 2 [Homo sapiens]
AAK57194.1	NADPH oxidase 5 delta [Homo sapiens]
XP_002825647.1	PREDICTED: NADPH oxidase 5-like isoform 2 [Pongo abelii]
XP_001085148.2	PREDICTED: NADPH oxidase 5 [Macaca mulatta]
XP_001495715.2	PREDICTED: NADPH oxidase [Equus caballus]
NP_001094607.1	NADPH oxidase5, EF-hand calcium binding domain 5 [Bos taurus]
XP_002722428.1	PREDICTED: NADPH oxidase, EF-hand calcium binding domain 5 [O. cuniculus]
XP_002920679.1	PREDICTED: NADPH oxidase 5-like [Ailuropoda melanoleuca]
EFB19538.1	hypothetical protein PANDA_009435 [Ailuropoda melanoleuca]
EFN89636.1	NADPH oxidase 5 [Harpegnathos saltator]
ABV44289.1	NADPH oxidase EF-hand calcium binding domain protein 5 [Ovis aries]
EGC36287.1	superoxide-generating NADPH oxidase flavocytochrome [Dictyostelium purpureum]
XP_001921894.1	PREDICTED: predicted NADPH oxidase 5-like [Danio rerio]
FAA00348.1	TPA: predicted NADPH oxidase [Apis mellifera]
NP_001093755.1	NADPH oxidase 5 [Gallus gallus]
NP_001097336.1	NADPH oxidase [Drosophila melanogaster]
FAA00347.1	TPA: predicted NADPH oxidase [Anopheles gambiae]
